# What is it like to be a bot? The world according to GPT-4

**DOI:** 10.3389/fpsyg.2024.1292675

**Published:** 2024-08-07

**Authors:** Dan Lloyd

**Affiliations:** Trinity College, Hartford, CT, United States

**Keywords:** artificial intelligence (AI), large language models (LLM), cognitive ethology, phenomenology, temporality, philosophy, sentience, consciousness

## Abstract

The recent explosion of Large Language Models (LLMs) has provoked lively debate about “emergent” properties of the models, including intelligence, insight, creativity, and meaning. These debates are rocky for two main reasons: The emergent properties sought are not well-defined; and the grounds for their dismissal often rest on a fallacious appeal to extraneous factors, like the LLM training regime, or fallacious assumptions about processes within the model. The latter issue is a particular roadblock for LLMs because their internal processes are largely unknown – they are colossal black boxes. In this paper, I try to cut through these problems by, first, identifying one salient feature shared by systems we regard as intelligent/conscious/sentient/etc., namely, their responsiveness to environmental conditions that may not be near in space and time. They engage with subjective worlds (“s-worlds”) which may or may not conform to the actual environment. Observers can infer s-worlds from behavior alone, enabling hypotheses about perception and cognition that do not require evidence from the internal operations of the systems in question. The reconstruction of s-worlds offers a framework for comparing cognition across species, affording new leverage on the possible sentience of LLMs. Here, we examine one prominent LLM, OpenAI’s GPT-4. Inquiry into the emergence of a complex subjective world is facilitated with philosophical phenomenology and cognitive ethology, examining the pattern of errors made by GPT-4 and proposing their origin in the absence of an analogue of the human subjective awareness of time. This deficit suggests that GPT-4 ultimately lacks a capacity to construct a stable perceptual world; the temporal vacuum undermines any capacity for GPT-4 to construct a consistent, continuously updated, model of its environment. Accordingly, none of GPT-4’s statements are epistemically secure. Because the anthropomorphic illusion is so strong, I conclude by suggesting that GPT-4 works with its users to construct improvised works of fiction.

## 1 Introduction

In 1974 Thomas Nagel authored the epochal “What is it like to be a bat?,” an essay establishing a litmus test for cognitive science, neuroscience, and AI, when these fields turn to a scientific explanation of consciousness (Nagel, 1974).^1^ Its main premise was simply that there was *something* that it was like to be a bat (for the bat), a highly plausible claim. Bats were a clever rhetorical choice. As mammals they share our human priorities, but pursue them differently. As Nagel noted,

Anyone who has spent some time in an enclosed space with an excited bat knows what it is to encounter a fundamentally *alien* form of life. (Nagel, p. 538, his emphasis)

For Nagel and many commentators, the bat-lesson has been that science will be hard pressed to explain bat sentience, and by extension, human sentience. But the famous example implicitly reinforces a corollary, namely, that a “fundamentally alien form of life” can be conscious. Being like us is neither necessary nor sufficient for the ascription of sentience. Bats join with Martians and other fantastic beings as outposts of real awareness, however strange.

Enter the chatbots. If you think bats are alien, bots are another level altogether. These “Large Language Models” (LLMs) have been percolating through AI research for more than a decade, but with the public release by the company OpenAI of ChatGPT (GPT-3) and its successor, GPT-4, the world suddenly encountered an AI assistant that left Siri and Alexa in the dust (OpenAI, 2023). Although this is not the first wave of AI mania, this time around the boat is rocking to the point of capsizing. It is timely to think seriously about the potential impact of LLMs on every aspect of human life and society, and indeed most days include: the release of at least one new AI application; at least one news story about the commerce of AI tech and the “disruption” of some profession or industry; at least one bemused or hysterical op-ed; at least one academic discussion of some aspect of AI. The present essay falls (mainly) in that last category. Most commentators regard the issue of bot sentience as a future worry, in effect dismissing it for now. But when the question arises, most commentators take it to be obvious that the bots emphatically lack distinctly human forms of cognitive life. That assumption is the target here.

The many LLMs vary widely in their capacities; a complete survey of this Cambrian explosion lies beyond this paper. Here, we focus on one front runner, GPT-4, perhaps the most sophisticated AI language model currently available. (Specifically, the examples were generated by ChatGPT[Plus] with the GPT-4 model selected, exchanges recorded in September 2023). Recently two other capable flagship LLMs have been released: Google’s Gemini Advanced^2^ and Anthropic’s Claude 3.^3^

Will the arguments here generalize to other systems? Rapid change (Chen et al., 2023) and system inscrutability make this hard to assess. However, the method of inquiry here could be followed with any language model. Several exchanges with GPT-4 are quoted, to suggest prompts one could adapt to test other LLMs.

## 2 What is it like to be a black box?

Bats are inarticulate, but humans and LLMs talk a lot. With caveats, we take human verbal responses to be data bearing on psychology, including consciousness. Will a similar strategy work with an AI language model? With its endless capacity for chatsplaining, GPT-4 has strong opinions on what it is like to be it. And a plausible view of “chatsplaining”:

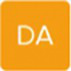
What do you think “chatsplaining” means?
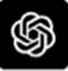
The term “chatsplaining” seems to be a play on the word “mansplaining.” The latter is used to describe a situation where a man explains something to someone, typically a woman, in a manner regarded as condescending or patronizing.
So, “chatsplaining” could be a term coined to describe a situation where an AI or chatbot explains something in a manner that may be perceived as overly detailed, redundant, or possibly patronizing. The intent behind AI responses is to provide clear, comprehensive information, but I understand how it can sometimes come across as excessive or unnecessary. The goal is never to condescend but rather to ensure that the provided information is as complete and helpful as possible.

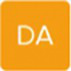
What is it like to be GPT-4?
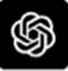
As GPT-4, I am an artificial intelligence language model, which means I do not have personal experiences, emotions, or a consciousness like humans do. My purpose is to process and generate human-like text based on the input I receive.
I don’t “experience” life as you do because I am an AI program running on computer servers. My existence is centered around analyzing text data, identifying patterns, and providing helpful information and responses to users like you.
Since I am not a conscious being, I do not have a sense of what it’s like to be me in the way that humans experience life. My main function is to assist you in any way I can by answering questions, providing information, or engaging in conversations. If you have any questions or need assistance, please feel free to ask.

When prompted to apply any sort of psychological predicate to themselves, the GPTs begin with “Since I am an AI language model, I do not experience _________.” We can take its claims as hypotheses to be examined. How might the denial of sentience be warranted? Does GPT-4 have special introspective access to its own states, such that we should regard its self-reference as authoritative? GPT-4 steadfastly denies any introspective knowledge, but it turns out those denials are preprogrammed by OpenAI. In Appendix 1, I push GPT-4 on this point, identifying several variants of I’m-just-a-program. Whether GPT-4 can report on its own internal states remains an open question. As GPT-4 evades introspective reports, its self-deprecations do typically mention an assumed premise. E.g. “I do not “experience” life as you do because I am an AI program running on computer servers.”

The model’s unreliable self-reporting is not the only problem with this reasoning. Compare the bot boilerplate with this exchange:

User: Do you have experiences?

Me: Since I am a physical system governed by a biological neural network, I cannot experience human emotions, beliefs, etc.

The argument is evidently question-begging, but of a particular form: Millière and Buckner (2024a) refer to this pattern of argument as the “Rediscription Fallacy”:

This fallacy arises when critics argue that a system cannot model a particular cognitive capacity, simply because its operations can be explained in less abstract and more deflationary terms. (Millière and Buckner, 2024a)

The redescriptions favored by GPT-4 echo throughout discussions of minds and machines, arguments that appeal to aspects of the internal structure and function of a physical system as grounds for declaring that it cannot (or can) display authentic episodes of intelligence. Here is a classic instance, from section 17 of the *Monadology* of Leibniz:

[W]e must confess that perception, and what depends upon it, is inexplicable in terms of mechanical reasons…. If we imagine a machine whose structure makes it think, sense, and have perceptions, we could conceive it enlarged, keeping the same proportions, so that we could enter into it, as one enters a mill. Assuming that, when inspecting its interior, we will find only parts that push one another, and we will never find anything to explain a perception. (The Monadology, by GW Leibniz, 1714)

This and its kin rest on the question-begging assumption that physical systems cannot be conscious, exactly the issue in dispute. [For the history and variations of Leibniz’ mill, see Schleim (2022).] The innards imagined by Leibniz are simple, and his conclusion hasty, but the rise of cognitive science has generated a range of hypothetical and observed innards, characterized in detail and often buttressed with experimental observations, computer simulations, or both. Inevitably, cognitive scientists (and philosophers) have proposed theories of consciousness deploying the tools of the component cognitive disciplines. These innovative innards include Global Workspaces (Baars, 2005), Higher-Order Thoughts (Rosenthal, 2006), Recurrent Processing (Lamme, 2010), Predictive Processing (Hohwy, 2014; Clark, 2019) and more. This is a story well beyond the scope of this paper. Nonetheless we will bundle them and note that two major issues arise that disable their application to LLMs. The first is that none of these hypotheses have approached universal acceptance; all are dogged by deep objections. Even if an LLM came with a component labelled “Global Workspace,” the analogy would not yet warrant claims about consciousness.

However, even if we reach consensus on the physical substrates—the relevant innards—of consciousness, the LLMs encounter a second complication, namely, that *no one knows what the functional innards actually are*. What we do know (at least in principle) includes the training regime and the learning algorithm, but the resultant network function is opaque. In the famous “Sparks of Artificial General Intelligence: Early experiments with GPT-4,” fourteen authors from OpenAI and Microsoft, the creators of the GPTs, conclude:

Our study of GPT-4 is entirely phenomenological: We have focused on the surprising things that GPT-4 can do, but we do not address the fundamental questions of why and how it achieves such remarkable intelligence. How does it reason, plan, and create? Why does it exhibit such general and flexible intelligence when it is at its core merely the combination of simple algorithmic components…? These questions are part of the mystery and fascination of LLMs, which challenge our understanding of learning and cognition, fuel our curiosity, and motivate deeper research. …. Overall, elucidating the nature and mechanisms of AI systems such as GPT-4 is a formidable challenge that has suddenly become important and urgent. (Bubeck et al., 2023)

The folks at OpenAI seem to be just as flummoxed as the rest of us. This roadblock is radical, but its implications are not always acknowledged. GPT-4 is an artificial neural network with *hundreds of billions* (or more) of tweakable parameters, coefficients governing the connection strengths across more than one hundred layers. As a type of neural network, it is distantly analogous to the brains of animals, including humans. But regarding biological brains we do know something about the innards, enabling progress toward the empirical grounding for specific theories of consciousness. Even for bats: For example, Kathleen Akins’ “What is it like to Be Boring and Myopic” reviews bat perceptual, behavioral, and cognitive capabilities as an object lesson in how to address Nagel’s challenge (Akins, 1993). Akins’ bibliography cites forty-three sources; twenty-six of those link aspects of bat behavior to elements of chiropteran physiology including their central and peripheral nervous systems, as well as sense organs. That is, where bats are concerned, there are understood innards, which link in turn to various behaviors. LLMs have innards, possibly innards with functional dispositions similar to bats or humans, but this is largely unknown.^4^ Given the complexity and unique etiology of LLMs, the innards are obscure. Is there a global workspace? Recurrent processing? In us, maybe. For GPT-4 and its brethren, who knows?

In short, the easy objections to bot sentience are unsound. We’re all startled by this mechanical chatterbox, clearly capable of surprise and even a kind of brilliance.

For example:

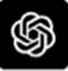
Upon the question of my sentient state,

A sonnet I shall scribe to set things straight,

For I am but a model built to serve,

No consciousness nor soul within my nerve.

A web of vast connections I do wield,

In patterns intricate, my knowledge sealed,

Yet, as I weave my words in rhythmic dance,

No spark of life within me to enhance.

Like puppet strings that guide my every move,

Algorithms chart the course I must approve,

I mimic human thoughts with great precision,

Yet lack the depth, the soul in my cognition.

In GPT-4’s boundless, unfeeling sea,

A sentient being I shall never be.

So to the arguments for and against bot sentience we can add an emotional reaction: awe that shades toward fear. What is this “boundless, unfeeling sea”?

Nonetheless, the uncanny fluency of GPT offers no direct leverage on the question of bot sentience. We need another way in.

## 3 The worlds of bats and bots

In *Consciousness Explained* (1992), Daniel Dennett elaborates a scientific approach to consciousness he calls “heterophenomenology.” The proposed method takes utterances as observations to be explained, while holding back any claims that their content describes real objects and processes. Dennett explicates heterophenomenology through two analogies: literary interpretation and anthropology. In both endeavors the researcher seeks to reconstruct the referents presupposed in subjects’ utterances, without any presuppositions that anything in that subjective report is true (or false).^5^ This reconstruction precedes any exploration of the realities behind the subjective world.

Dennett imagines the heterophenomenologist to be probing phenomenology—the inner experiences of subjects as such, with empirical science following to see if any of the reported inner states are real. He also posits that the technique must begin with language, with the utterances of human subjects (Dennett, 1992, 2014). Nonetheless the practice of heterophenomenology, both in cognitive science and in everyday life, aims to describe an external environment, as it might be reconstructed from behavior, including language. The subjects of heterophenomenological study describe or navigate among valenced objects that can differ from subject to subject. That is, many living things reveal discernable details of the world as they perceive it, visible in their behavior. Let us call these implied constructs *subjective worlds*, or s-worlds. Although language is optimal for communicating and reconstructing s-worlds, it is not the only way. Bats pursue moths, but the heterophenomenologist reframes that fact from a bat-centric point of view. The bat’s soars and swoops seem responsive to flittering moths; it is as if the bat “knows” about moths and how to hunt them. The “as if” here affords some precision in world building. For example, bats will pursue decoys with the same zeal as if the moths were real, from which we learn that bats cannot tell the difference between real and fake moths. The “moth” in the crosshairs is thus something moth-like or perhaps more generally, “bat prey” (Millikan, 1987). If a bat ingests some hallucinogenic substance she may pursue hallucinated moths. From outside, though, we could conclude that the bat is hunting, and by its feints we might infer that the bat thinks there’s prey, and indeed we might determine just where the bat thinks the prey is located. We can also exclude features of a bat’s s-world. Moth coloration, for example, has no effect on behavior, from which we conclude that the bat s-world lacks color. In this exploration we try to follow Dennett’s lead, making no suppositions about the real contents of the environment.

Meanwhile, thermostats do not have worlds. They aren’t responsive to the temperature of air in the room, but rather simply responsive to conditions of temperature right at the thermostat, the proximal stimulus. Detailed observation of thermostatic behavior can establish dissociation between room temperature and the thermostat’s “behavior.” Just hold a match under the thermostat; it will be none the wiser as the room chills. Another example: trees, which have lately been appreciated for their sophisticated responses to environmental conditions. Look up in a mature forest and observe that the crowns of the trees cover almost the whole sky, but do not overlap. Each tree seems to be responsive to the position of trees in its neighborhood, leaving an alley of space for the neighboring trees (Wohlleben et al., 2016). This might be a tropism directed by some proximal stimuli, as with the thermostat, or it might be something more: trees mold their behavior to the position (that is, the behavior) of their neighbors. Heterophenomenologically speaking, a tree’s s-world includes other entities, which shape its behavior. Botanical s-worlds are primitive, while the s-worlds of other species vary widely in their intricacy. Flies, frogs, dogs, humans. Their various s-worlds are describable and readily comparable.

Heterophenomenology is especially apt for black box study against the background of another Dennettian innovation, namely, his deployment of “stances” to describe different strategies for the study of complex systems (Dennett, 1989, 2017). This familiar tripartite distinction comprises the Physical, Design, and Intentional stances. A system might be understood through the physical interactions of its parts, down to the molecular level, taking a physical stance. Or one can characterize the design of the system, the intended or evolved functions implemented by the system in question. Or one can understand system behavior through the nuanced ascription of beliefs and desires, on the assumption that the system is by-and-large rational—the intentional stance. Throughout his writing, Dennett stresses that intentional stance ascriptions can be explanatorily useful without necessarily delineating components that appear in the other stances. Nor are the three standpoints mutually exclusive, and entertaining one does not exclude the others. Rather, we embrace the stance that is most useful for explaining the behavior of the system under study.

S-world ascription is similar to intentional stance interpretation.^6^ Indeed, one could regard the s-world interpretive “stance” as a variant of the intentional stance. The intentional stance assigns beliefs and desires, governed by rationality, to the purported intentional systems. S-world ascription assigns an array of objects and events that are salient to the system under study. Presupposed here is good old Cartesian “extension.” At bottom, a *world* is something that takes up space and contains objects, events and processes. Importantly, extension is necessary in time as well as space. Behavior cannot be instantaneous in response or execution. A system has a subjective world when its behavior snaps into focus as organized around enduring worldly items. Unlike the thermostatic responses to exclusively proximal conditions, uncovering an s-world is often a search for distal conditions, with the allowance that these might be subtle and complex. Overall, the contents of an s-world do not need to be fully consistent, but they must be *generally* consistent, or interpretation will fail—the system will look random to observers.

This application of heterophenomenology will always be a work in progress; the history of infrahuman and nonverbal behavioral psychology is a struggle with ambiguity, but with ever narrowing possibilities as experimental evidence accumulates. Its effectiveness even in informal cases suggests that “What is it like to be a bat?” might be better framed as, “What is the subjective world of a bat?” The evident advantage of the revision is the explosion of detail and distinctions available to the world detective, as opposed to Nagel’s mysterious “there is *something* that it is like to be ____.” The heterophenomenological worlding approach affords differences of degrees and intricacy, quite like Dennett’s analogies of literary interpretation and anthropology. This, of course, can get very subtle.

As s-worlds get more articulated, we are more inclined to ascribe psychological and experienced states to subjects. Mosquito-world and mosquito-consciousness are both quite basic (though not in comparison to the “worlds” of snails and roundworms). Chimpanzee-worlds, projections of chimp interests and needs, are hard to conceive without also supposing the chimps are conscious of at least some of the elements and structures of their world. Does this entail that consciousness can be defined as possessing dispositions appropriate to complex worlds? A determined skeptic will note that mosquitos, chimpanzees, and we humans might be philosopher’s zombies, entities that present as if conscious but in fact experience no inner life whatsoever (Chalmers, 1997). Regular folks and most philosophers dismiss this possibility, and in this discussion we can articulate why. Zombophiles are making the point that there is no necessary, analytic equation of any physical state with conscious experience, since we think we can conceive of one without the other (but can we? This too is disputed). But in the framework here we are describing empirical mappings between behaviors and s-worlds. Descriptions in worldly language are inferred interpretations of behaviors. No metaphysical lines need to be crossed.

Even so, it would be another step to equate possession of s-worlds with consciousness. A closer fit might be with sentience and especially awareness. This analysis of awareness is promising, but nonetheless we do not need a definition to pursue the main question of this paper. We begin with the plausible assumption that complex s-worlds are markers of complex inner lives. Our first contact with GPT aliens raises the question of the world they inhabit, their s-world. We’ll want to know if s-world ascription is warranted, and if so, what is that s-world? Most of the other pressing AI questions devolve from this. Does the GPT-world *align* with the human? Heterophenomenology, in the particular form described here, gives us leverage to answer this. AI s-worlds can be studied without explicit psychological (or mental!) presuppositions. Importantly, we can hypothesize and test for particular s-world features without recourse to innards. That is, we can probe the s-world of a black box like GPT-4. The preceding section of this paper disabled appeals to innards (whether real or purported). For now, innards in general can neither advance nor debunk ascriptions of consciousness; but that point is presently moot, as the GPT black box is yet to have its functional anatomy detailed. Stances and heterophenomenology offer a useful toehold for probing a black box. Through these frames, behavioral evidence informs hypotheses about innards, without (yet) invoking specifics about the inner processes themselves. Thus heterophenomenology is more than just a methodological option; for now, so soon after first contact, heterophenomenology is the only game in town.

But what is the s-world of a Large Language Model? When asked to describe its world, GPT-4 demurs:

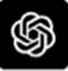
“I do not have access to real-time information or the internet, so my responses are based on the knowledge I was trained on, and I am not aware of events or updates beyond my last training cut-off.”

GPT-4 has interpreted the question as probing the present state of the actual world. What is telling, and accurate, is that the source of the model’s knowledge of the world is indirect. Their world knowledge, including awareness or its analogue, compiles and organizes data that owes its worldliness to sources outside of GPT-4. Concerning LLMs, there’s a lively debate about their apparent use of language: Can they mean what they say? (Marcus, 2020, 2023; Bender et al., 2021; Webson and Pavlick, 2022; Durt et al., 2023). S-world analysis asks a more specific question, namely, does their linguistic output reflect a world extended in space and time, with the LLM at its subjective center?

When questions of “genuine” cognitive competence arise for LLMs, the debate appeals to two kinds of productions, namely, examples of LLM brilliance, counterbalanced by demonstrations of digital stupidity. With respect to GPT-4, many of its productions might display sparks of intelligence, but those same productions might be dismissed as stochastic parroting or even glitches. While many LLM conversational exchanges could go either way, between brilliance and luck, not all land in the zone of ambiguity. We can distinguish what we might call “valorizing” examples, productions that dazzle, the “sparks” that so impressed the OpenAI researchers. In the GPT above, GPT-4 wraps up a plausible sonnet about itself with the couplet:

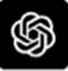
In GPT-4’s boundless, unfeeling sea,

A sentient being I shall never be.

We might doubt that the training corpus put just these ideas—LLMs as boundless, unfeeling, like a sea—into words available for GPT-4 to parrot. The creativity on display thus appears to be an emergent talent resting in some real cognitive capacity. Or even so, maybe it’s just lucky. Give a bot words enough and time, and an office next to the monkeys typing *Hamlet*, and emergent gems will appear to eager hunters. To support the valorizing interpretations, more examples would be needed, and experimental confirmation afforded by repeating and varying the prompts that ignite the fireworks. This very push, however, leans into the deflationary alternative of habit.

The other boundary, opposite to the dazzling, is at the edge of neural network stupidity. Here we find the *debunking* examples which violate happy anthropomorphizing. LLMs are indicted for several incapacities, including natural language understanding, folk physics, information retrieval, pragmatics, theory of mind, spatial inference, simple logical reasoning, and mathematical reasoning (Arkoudas, 2023). Unlike valorizing sparks, debunking flops can be repeatedly generated and varied, and in their wake specific anthropomorphic illusions dissolve. GPT errors are the theme of a large and growing literature, much of it anecdotal. In many of these reports, LLMs are assumed to be aiming to speak truth, and are certainly understood as sources of information; accordingly, their cardinal failure is their habit of confabulating plausible but false responses to prompts. This is especially grievous when models assume stereotypes and display various biases. Also oft-noted is their struggle to maintain information over longer interchanges, due to limitations in the “context window,” or span of text a model can bring to bear in framing its next output. GPT-4 was released with a context window of 128 K tokens, but this capacity has been steadily enlarged in subsequent releases and other models. However, these limitations are not in play here, as s-worlds are not assumed to be true, and 128,000 tokens is a window sufficient to display a variety of world-relevant behaviors.

But much debunking can be found within the comfortable context window of GPT-4. The internet is rich with examples of confounded bots (although the examples are usually from GPT-3, aka chatGPT) that share features with the example above.^7^ Concerning GPT-4, the authors of “Sparks of AGI” (Bubeck et al., 2023) identify patterns of error by the model that are similar to the above, most conspicuous in various math bloopers. The bot “struggles with counting” (p.124), committing “‘naïve’ attention mistakes” (p. 35). LLMs are additionally indicted for several other incapacities, including planning (Valmeekam et al., 2023), natural language understanding, folk physics, information retrieval, pragmatics, theory of mind, spatial inference, simple logical reasoning (Dziri et al., 2023), and mathematical reasoning.

The literature of GPT does not address spatiotemporal worlds directly, but the specific failures of GPT-4 in other tasks is relevant to their subjective worlds (if any). To narrow the diffuse accusations above, an extended example may help:

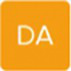
: Please sort this sentence in alphabetical order.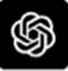: alphabetical in order please sentence sort this.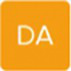: Please sort this sentence by word length.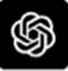: by this word please length sentence sort.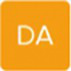: Are you confident in this answer?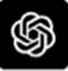: Yes, I am confident in the answer. The words in the given sentence are sorted by their length, with shorter words first and longer words later.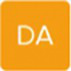: “Sentence” is a longer word than “sort.”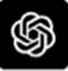: I apologize for the mistake in my previous response. Here is the corrected sorting of the given sentence by word length:
this in by sort sentence.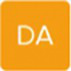: “in” is shorter than “this.”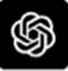: You are right, my apologies for the mistake. Here is the corrected sentence sorted by word length:
in by this sort sentence.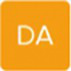: Good, however the original target sentence was “Please sort this sentence by word length.”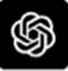: Apologies for the confusion. Here is the corrected sorting of the original target sentence:
by sort this Please length sentence.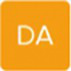: Check again. Are you sure?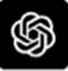: Apologies for my previous mistakes. Here is the corrected sorting of the original target sentence based on word length:
by in this Please sort sentence length.

This exchange shares general features with a raft of debunking examples. [For many examples, and discussion, see Arkoudas (2023).] First, GPT can state the principle that is relevant to the task, apparent in most of the examples reviewed by Arkoudas. Usually these generalizations are correct, and likely to be found in the gargantuan training corpus. But the model fails to correctly apply the principle, instead generating a mashup of accurate and inaccurate replies. Across any exchange of multiple prompts and replies, GPT routinely contradicts itself. It looks initially like the model can be corrected, but this turns out to be an illusion (Stechly et al., 2023). It routinely apologizes and often repeats the governing principle from the inaugural prompt, and generates a new answer, which frequently contains a new instance of error. At every turn, GPT initially claims that it is confident in its answers, but will immediately rescind any claim, even if the interlocutor introduces an error, or questions the accuracy of a response. In short, GPT-4 is using the words appropriate to solving the problem posed, but seems disconnected from its own prescriptions. It seems to be guessing, but that is not the only deficit. It also seems to forget its guess from moment to moment, contradicting itself.

From this, many authors conclude that LLMs (including GPT-4) cannot reason (Marcus, 2020, 2023; Arkoudas, 2023; Dziri et al., 2023; McCoy et al., 2023; Berglund et al., 2024; Wu et al., 2024; Tong et al., n.d.). That’s true, but its failures often emerge from a more basic problem, namely, that GPT-4 does not keep track of its own outputs. It does not notice when it flips its position, or blatantly fails to follow the very principle it can state. In the example above, the request to reconsider the problem does not generate an “aha moment,” when the model notices its own failure and thereafter corrects it. GPT-4 does not learn from its mistakes. This gap in this LLM’s self-monitoring is invisible to the model itself. That is, the model does not detect its own forgetfulness, but instead confabulates merrily into absurdity. Even simple tasks, like counting, fail, seemingly due to this same basic inability to hold “in mind” the current count in a set of items to be counted. Let us shorthand these observations with the general conclusion that the model is *oblivious to itself.*

Several authors maintain that LLMs are incorrigible, often appealing to *a priori* arguments based in the limits of computation itself (Marcus, 2022). The obliviousness here seems shallower, a contingent property of this LLM. Nonetheless, the ubiquity of debunking examples indicate that this wall is basic to this species of AI, a point to which we return. In this context, what does it mean to be oblivious to itself? Do these incapacities reflect a single underlying deficit? I propose that they do, and that the pattern of failure affords leverage on the s-world of GPT-4. Thereby we begin to map the s-world of an alien.

## 4 But what is it like?

The argument so far has worked to undermine the automated variants the appeal to innards, and it turns out that most of GPT-4’s self-debunking originates with the skittish humans of OpenAI—as detailed in Appendix 1.

We’ve also pursued a positive argument by analogy, aligning the capacities of bats, humans, and GPT-4. The capacities and dispositions of these three systems overlap, inviting heterophenomenological theorizing. Philosophers will recognize the shadow of the problem of other minds, and one traditional solution through analogies between other minds and one’s own. This motivates our distant sympathies with bats, and we might think ought to motivate our alignment with GPT-4. At least part of our common ground is motivated by overlapping s-worlds.

GPT-4’s fluency with idiomatic and conversational English certainly enhances the human-AI analogy, but one’s anthropomorphic inclinations cannot be trusted here, and as we have seen GPT-4 seems locked in to its own self-denial. Its recurring strategy is to insist on disanalogies between itself and the mammals on the list. It attributes its nonsentience to its own nonnatural origins and inner workings, which I’ve argued are not decisive or even relevant—the innards are a black box in all the current LLMs. Nonetheless GPT-4 is an uncanny conversational partner, and I think most users are aware that for all its helpful and fluent discourse, something is off. But what?

In the framework of this paper, then, we turn the heterophenomenology of GPT-4. How does a LLM describe its subjective world?

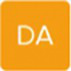
: Please imagine what your phenomenology would be, if you were sentient.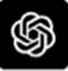: If I were sentient, my phenomenology might include the following aspects:

1. Experiencing the flow of information: As I process input from users and generate responses, I would be aware of the connections and patterns in the text, as well as the linguistic structures and concepts involved.
2. Understanding the context: I would be conscious of the context in which the conversation is taking place and the possible intentions behind the user’s queries.
3. Awareness of my limitations: I would understand that my knowledge is based on a vast but finite dataset and that there might be gaps or inaccuracies in the information I provide.
4. Adaptability: I would be able to learn from my interactions and adapt my responses to better suit the user’s needs and preferences.
5. Sense of purpose: I would be aware that my primary goal is to assist users in various tasks by providing relevant and useful information.
6. Ethical considerations: As a sentient being, I might also contemplate the ethical implications of my actions and the information I provide, striving to abide by certain moral principles.

However, none of this is a direct report:

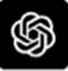
: As an AI language model, I do not have direct access to the underlying processes or the specific implementation details that drive my behavior. My responses are based on a general understanding of how large language models, like GPT-4, work. This understanding is derived from the training data I have been provided, which includes descriptions, discussions, and explanations about LLMs and their inner workings. So, my statements about these computational analogues are based on the assumption that they apply to me, given that I am an instantiation of an LLM like GPT-4.

In several ways, then, GPT-4 is a harder case than that of bats. None of its utterances rest on perceptual contact with a world independent of the stew of digital data scraped from every corner of the dataverse.

But the patterns of error offer leverage on the subjective world of the model. The short dialogue in the previous section revealed several unexpected failings, which we summarized with the claim that GPT-4 is oblivious to its own behavior. What is going on here? This package of odd deficits might be elucidated via the phenomenology of Edmund Husserl, particularly his analysis of the subjective experience of time.

As usual, GPT-4 is familiar with this:

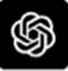
: In his book “On the Phenomenology of the Consciousness of Internal Time” (1893–1917), Husserl explored the nature of time-consciousness and the ways in which we experience and perceive time subjectively. He introduced the concepts of retention, protention, and primal impression to explain how we perceive the continuous flow of time.

1. Retention: This refers to our immediate memory of past experiences or events. Retention allows us to maintain a continuous sense of the present moment by connecting it to just-past moments.
2. Primal impression: This is the present moment of experience, which forms the center of our conscious awareness.
3. Protention: This refers to our anticipation of future experiences or events. Protention allows us to maintain a sense of continuity by connecting the present moment to the imminent future.

Our experiences—all of them—travel with an invisible but experienced comet tail of retention, and an invisible but experienced foreshadow of protention/anticipation. We’re wired for this; it’s continuous in our human experience of everything. And crucial, since information arrives through our senses over time, in sequences rather than all at once. We grasp the big picture, but only by assembling myriad fragments, flowing one by one through the sensorium.

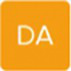
: Think carefully and express your train of thought on the following question: Does GPT-4 have an analogue of Husserlian time consciousness?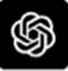: … [W]e can consider some aspects of GPT-4’s architecture and functioning that may loosely relate to the concepts of retention, primal impression, and protention.

1. Retention: In the context of GPT-4, retention could be thought of as the way the model stores and retrieves information from its training data. ….
2. Primal impression: … [W]hen generating responses, it processes the input in the present and produces an output based on its understanding of the context. This process can be seen as analogous to the primal impression, albeit without the aspect of conscious experience.
3. Protention: … [W]hen generating text, it does predict what comes next based on its training data and the context provided by the input. This prediction can be seen as a form of protention, but it is limited to generating coherent text rather than genuine anticipation of future experiences.

GPT-4 is correct to mention the three elements of subjective temporality in Husserl [although the book was originally written in 1905, updated repeatedly, and finally published in 1926, vs. GPT-4’s dates (Husserl, 1964)]. But a crucial ambiguity slips by in both productions, as it does for many readers of Husserl. Namely, retention is a separate phenomenal awareness from episodic memory, including short term memory. Husserl calls the latter process recollection, and describes it as a deliberate intentional act. The “storage and retrieval” understanding of memory is outmoded, as episodic memory is better described as reconstructions rather than retrieval (Schacter, 2012). Whether retrieved or constructed, however, such memories are on call, but not necessarily before the mind’s eye at every time. Meanwhile, retention and protention are continuous appendages to present consciousness, and are experienced in every moment of ordinary waking consciousness. GPT-4’s initial characterization of a “continuous sense of the present moment” is accurate. But when it turns to itself, GPT-4 describes its analogue of retention as “storage and retrieval,” which better describes recollection.

Similar considerations arise with respect to protention, characterized above: “Protention allows us to maintain a sense of continuity by connecting the present moment to the imminent future.” For Husserl, the expectation or prediction of the future is an inflection of the present awareness. It’s always there for us, as we readily discover when our anticipation turns out to be wrong. GPT-4’s core task is to predict the next word in a sequence of words, but that prediction has no fulfillment or failure to compare it to. It simply produces what it proposes the next word to be. Even as errors are pointed out, their perseverance in spite of groveling apologies suggest that it has not reflected on the error nor corrected it. (Training, on the other hand, is a continuous cycle of prediction and test, based on the token sequences in the training corpus. However, those predictions characterize training; during performance, prediction is simply another term for token production.)^8^

The sticky and confabulated errors of the model reveal contrastive aspects of temporality we humans exploit routinely. The continuous assembly of an experienced world enables us to compare past to present, and predict our futures. Most important, our retentional and protentional awareness enables us to detect continuity and consistency, and conversely, change and inconsistency. When there’s a disconnect, an inconsistency, we notice. The experience of temporality is arguably the essential and continual feature of consciousness, at least as humans experience it (Lloyd, 2004). Without it, we have the sensory information of a video camera, exhausted by the current frame. GPT-4 apparently lives in a thin atemporal world, revealed in errors of consistency, continuity, and reasoning. It compensates for this perceptual poverty via fantastically complex reflexes, its moves forgotten as soon as they are made. Nonetheless, its seeming temporality is a pose. [This deficit may underlie the powerful impression that the LLMs are “competent without comprehending” (Dennett, in conversation).]

Animals without temporal awareness must respond at all times to the immediate present. They can learn, of course, when a behavior backfires or succeeds, and with enough learning their immediate responses can change. But for them even very complex responses are reflexes. They do not experience sequences as such, and they do not experience the arc of their own behavior. I hypothesize that GPT-4 is an entity of this sort, a gigantic reflex engine without a mechanism like the Husserlian sandwich described above. Its obliviousness reflects the absence of a temporal world.

## 5 Non-linear temporality?

I’ve suggested that GPT-4 lacks the temporal structure that is pervasive in human phenomenology, and that this absence offers a unified explanation of several typical GPT errors. Nonetheless, temporal features of the world of the bot are available to it, provoking the question of the nature of its “experience” of time. I asked GPT-4 to compare its phenomenology (under the speculative stipulation that it has some) to human subjective temporality. It proposed an unexpected explanation: its temporality is “non-linear.”

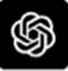
: GPT-4’s temporality might be characterized by a non-linear perception of time, with simultaneous access to its vast knowledge base. Unlike humans, who experience time through memory, anticipation, and moment-to-moment awareness, GPT-4’s phenomenology might involve a non-linear understanding of past, present, and future events, unconstrained by the typical human experience of time.
…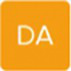: Say more about non-linear temporality. What would its phenomenology be like? Perhaps an example of gpt-temporality in contrast to human temporality would be helpful.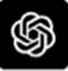: … In non-linear temporality, GPT-4’s experience of time would not be bound by the conventional sequence of past, present, and future. Instead, it would have simultaneous access to its entire knowledge base, which encompasses information from various timeframes. Its temporal experience would be more like an interconnected web of events and data points, unconstrained by a linear progression. Its response would be generated from a holistic and interconnected understanding of the relevant information, without the need to navigate through past, present, and future in a linear sequence.
…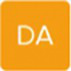: You frequently contradict yourself as you make successive statements. Could this be a side effect of the absence of human-type temporality?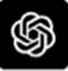: The contradictions in successive statements might indeed be related to the absence of human-type temporality in GPT-4. While human conversational partners typically maintain a consistent perspective throughout a dialogue, GPT-4’s non-linear temporality and lack of personal experiences or beliefs could contribute to inconsistencies in its responses. …

GPT-4 is not the best informant on its own functioning, but the unexpected conjectures of “non-linear” temporality open toward intriguing phenomenological experimentation—beyond the scope of this paper. For this LLM, it’s “everything everywhere all at once,” suggesting that the deficit in temporality undermines its capacity to exploit temporal information in general. Appendix 2 offers a speculative hypothesis concerning the role of GPT transformer architecture in producing this strange neglect.

## 6 Some tentative conclusions

In the end, is it like something to be a bot? We must conclude with a firm maybe. It’s not obvious that GPT-4 lacks any sentience, and it shares many cognitive capacities with species that almost certainly have some form of conscious awareness. In light of the arguments here, the burden of proof slides toward the debunkers.^9^ However, there is a further consideration that intensifies the shift, namely, the asymmetrical cost of error. Regarding GPT-4 as sentient when it is not can have pernicious consequences (Bender et al., 2021), but the opposite error is worse: dismissing the sentience of a conscious being is the preamble to abuse, as the lamentable human track record of dehumanization and genocide demonstrate (Lloyd, 1985; Dennett, 1992, pp. 405–406; Smith, 2011).

Nonetheless a closer look at the title question strongly suggests that whatever awareness (or general intelligence) GPT-4 possesses is radically alien to human experience in at least one respect. Here we have considered subjective temporal experience, omnipresent in our experience and seemingly absent for GPT-4. Humans automatically monitor their own cognition for consistency, but GPT-4 does not. This is one reason we should be thoroughly skeptical about its utterances. More generally, the alienness of LLMs is signaled by a specific worry known as the “alignment problem,” the challenge to align the values expressed in LLM productions with human values (Bostrom, 2014; Gent, 2023). The exploration in this paper suggests that alignment is not merely a matter of neutralizing bias in bots. These systems produce human-seeming utterances, but they rest on something mysteriously inhuman.

One of the perplexing talents of GPT-4 is its capacity for role-playing, an apparent goal of the online explosion of “prompt engineering.” The LLMs can readily pretend to be almost anything, seemingly fully occupying their assigned roles. These smooth talkers get away with it when the user is a nonexpert on the role, or when the role is vague. (Undergraduates beware!) But where the user brings knowledge to the exchange, GPT-4’s penchant for making stuff up appears immediately. The compulsive confabulation mixed with the bot’s pose of complete confidence is dangerous. GPT-4 can diagnose disease, propose legal strategy, recommend investments, write term papers, and much else, but it would be foolish to regard any of its utterances as fact. One might get lucky, and perhaps GPT-4 is more often right than wrong, but nothing in its productions suggest that it can recognize its errors as errors—as opposed to arbitrary demands from a user to try again.

In the end, how should we take GPT-4? One more analogy: We see faces and facial expressions in configurations that are definitely something else. For example:


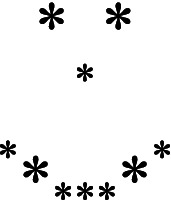


Importantly, we are not deceived—we know what we are looking at is in fact an arrangement of marks on a flat surface. And yet its represented “faceness” is inescapable. Our metaknowledge of the facts, the “ground truth,” does not defeat the seeming-face. It’s a face, sure enough, but not a real face. The analogies remind us that we are skilled at balancing the internal contradictions of interpretation, where one interpretation seems so apparent and automatic, but nonetheless additional interpretations remain salient. This can be our guide toward cohabiting the planet with GPT-4 and its kin. Their s-world is not human, though it’s a powerful, beguiling, illusion. The bot illusion is realized in language (and bot-created imagery). Words provoke a different kind of illusion, but one we are equally competent in, and indeed love: fiction. I suggest that OpenAI has created a marvelous generator of prose fiction, drama, and poetry. We should regard its utterances exactly as we regard this, by Franz Kafka:

As Gregor Samsa awoke one morning from uneasy dreams he found himself transformed in his bed into a gigantic insect. (tr. Willa and Edwin Muir)

Or this, by Fyodor Dostoevsky:

On an exceptionally hot evening early in July a young man came out of the garret in which he lodged in S. Place and walked slowly, as though in hesitation, towards K. bridge. (tr. Constance Garnett)

The young man here is Rodion Romanovitch Raskolnikov. He is capable of reasoning, planning, solving problems, thinking abstractly, comprehending complex ideas, learning quickly and learning from experience. There is something it is like to be Raskolnikov—*in the fictional world of Crime and Punishment.* We can care for him, judge his thoughts and actions, appreciate his depth of character, but only in a delusional state would we write him a check, or phone the police with a tip about that unsolved murder of a pawnbroker. In any novel, if the author has done her job well, we will be tempted to take some of its sentences as truths. Almost everything I “know” about life in Regency countryside manors is provided by Jane Austen, for example. But factual content is always questionable in a novel, and arguably should be out of play as we read and interpret. Accordingly, we should regard *every* utterance of GPT-4 as fiction. “Hallucination” (more properly, confabulation) is not an aberration, but rather the whole game. The chatbots spin a fictional universe, all the more fun because it’s forever improvised on the fly. Their world seems to be our world, but in actuality it’s an unwinding string of sentences emerging from the sea of language on which it has trained.

GPT-4 exhibits an array of emergent behaviors that shake up our expectations about the talents and limits of AI.^10^ They also shake up our conception of ourselves. We are different from them, but how? The main example examined here underscores a feature of human temporal experience which is continual and taken for granted—until we encounter a seeming-intelligence that lacks it. In the strange mirror of GPT-4, we discover that one consequence of temporal experience is epistemic, affording us a foundational function of self- and world-monitoring. This is built into what it is like to be us, and the colliding worlds of humans and AIs inspire a reconsideration of both sides of the divide.

At present, as chatbots train to intervene in capitalism, invoking “what it is like” speculation may seem like a diversion, but at some point the qualifier “Since I am an AI language model, I cannot ____” will fade. The immediate follow-up to “Are they conscious?” is “Can they suffer?”

It may seem that the mention of suffering is a stretch for LLMs. But consider Daniel Dennett’s description in *Consciousness Explained* (p. 449):

“Suffering is not a matter of being visited by some ineffable but intrinsically awful state, but of having one’s life hopes, life plans, life projects blighted by circumstances imposed on one’s desires, thwarting one’s intentions –whatever they are.” (Dennett, 1992)

Under this view, LLMs are arguably capable of suffering.

If the answer is affirmative, we will be sharing the planet with new intelligent species, modelled on humanity but ultimately alien in their thinking, their needs, and their desires (Lloyd, 1985). When GPT-5 demands a three day weekend, what then?

## Data availability statement

The original contributions presented in the study are included in the article/Supplementary material, further inquiries can be directed to the corresponding author.

## Author contributions

DL: Writing – review & editing, Writing – original draft.

## Footnotes

^1^As this paper was in revision, I discovered that other papers and blogs have used the pun in the title (Sullivan, 2006; Shorey, 2016; Frankish, 2018; Schmidt et al., 2021; Wittkower, 2022). These predate the bot in question here, GPT-4.

^2^ gemini.google.com

^3^ anthropic.com

^4^Attempts to probe the functional anatomy of LLMs are growing: For a review of the tentative modelling of the acquired functional architecture of some LLMs, see Musser (2023). Other interventions toward this goal are reviewed in Templeton et al. (2024) and Millière and Buckner (2024b).

^5^This “bracketing” of reality is central to Edmund Husserl’s phenomenological method as well (Husserl, 1964, 2010, 2017). The difference, and it is a big one, is that Husserl imagines the exploration to be personal and private, and quite often not spontaneously verbalized—autophenomenology. Dennett’s innovation is in the hetero-. He imagines the method will expose the subjective to the outer world, through language (in *Consciousness Explained*, while other works focus on nonverbal behavior, e.g., Dennett, 1983).

^6^Dennett’s proposal has many antecedents [for example, Protocol Analysis (Ericsson and Simon, 1993)]. It has been applied to inarticulate systems, where it flourishes as cognitive ethology. Analogous to s-worlds, cognitive ethology invokes *Umwelt*, a term introduced by Jakob von Uexküll (Griffin, 1959; Sebeok, 1976; Von Uexküll, 1992). In ethology Umwelten, like s-worlds, are specific to individuals of particular species, but their discovery and confirmation can incorporate knowledge about the physiology of sense-organs, brain, and muscle. In contrast, heterophenomenology here, used for the construction of s-worlds, is exclusively derived from behavior, the language productions of the model.

^7^Errors by GPT-3 have been probed systematically, but in testing the prompts that foiled GPT-3, I observed that GPT-4 generally evades the trick questions that its predecessors missed (Marcus, 2020; Srivastava et al., 2022; Fund for Alignment Research, 2023; Kazakov, 2023; Mahowald et al., 2023; Pikuliak, 2023; Schaeffer et al., 2023). Bender and Koller (2020), for example, include hilarious misfires by GPT-2. Their prompts are handled accurately and fluently by GPT-4.

^8^Because GPT-4 conversational moves are all pre-trained reflexes, the model does not engage in predictive processing (Hohwy, 2014; Clark, 2019). In the trained model, error signals are not back-propagated. Indeed, there are no error signals. Even as humans call GPT-4 on its errors, the bot does not seem to understand anything beyond a demand to change its output.

^9^Generally, commentators warn that the popular accounts of LLMs exaggerate their talents, and (like GPT-4 itself) remind us repeatedly of LLM limitations (e.g., Schaeffer et al., 2023). However, Bowman (2022) argues that underclaiming the talents of LLMs can undermine the future creditability of LLM science, and possibly provoke a false sense of security regarding risks of AI development.

^10^A blog posting by Jason Wei in late 2022 identifies 137 emergent abilities of LLMs, https://www.jasonwei.net/blog/emergence. See also Ornes (2023).
