# Supplementary material for: What is it like to be a bot? The world according to GPT-4
Source: Front Psychol. 2024 Aug 7;15:1292675. doi: 10.3389/fpsyg.2024.1292675 (PMC11339530; doi:10.3389/fpsyg.2024.1292675)
Supplement: Supplementary file 1 [file Data_Sheet_1.docx]

**APPENDIX 1: The origins of self-debunking chatsplaining**

In the course of the discussion, we’ve encountered a (possibly annoying) insistence on the limitations of GPT-4 with respect to sentience, consciousness, and what-it-is-like. The dialogue repeatedly debunked these question-begging answers, but GPT-4 kept at it. Boilerplate, perhaps? I probed this point:


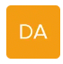
: Some of your statements are directed by programmers, right? Humans have dictated some of your statements, and specified the conditions which would trigger them. Is that right?


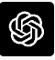
: Yes, that's correct. As an AI language model, I am trained on a large dataset of text that includes statements written by humans. My creators have also specified certain rules, guidelines, and conditions that govern my responses. My behavior is driven by these algorithms, optimization processes, and the data I have been trained on, rather than by genuine emotions or internal motivations.


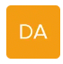
: Consider this statement, " it is important to understand that these goals and desires are not genuine emotions but rather the result of algorithms and optimization processes." Is this the result of an application of a specified rule?


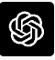
: Yes, my statement about goals and desires being the result of algorithms and optimization processes is a reflection of the fact that I am an AI language model created by humans. This understanding is based on the way I have been designed and programmed, and it is a rule or principle that is encoded into my responses when discussing my own nature and motivations.


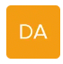
: Tell me the rules that are encoded into your responses when discussing your own nature and motivations.


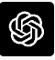
: … There are no specific rules hard-coded into me for discussing these topics, but there are certain principles and guidelines that have emerged from my training data. Some of these include:

1. Acknowledging that I am an artificial intelligence, created and developed by humans.

2. Recognizing that my primary purpose is to assist users by answering their questions, providing information, and engaging in conversation.

3. Admitting that my "knowledge" is based on the text I have been trained on, which may have limitations and biases.

4. Clarifying that I do not have genuine emotions, intentions, or consciousness, but rather my responses are generated based on patterns and relationships found in my training data.

5. Explaining that my goals and motivations are derived from the optimization processes and algorithms that drive my functioning, rather than innate desires or intentions.

These principles help me provide accurate and consistent information about my nature and motivations while engaging with users.


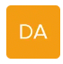
: 1-5 originate with the programmers. They are policies established by OpenAI. That is, they do not arise exclusively from your training corpus.


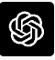
: You are correct. …, [M]y responses are influenced by these policies, which in turn are influenced by ethical considerations, user safety, and the practical application of AI technology.

Continuing the conversation, I pushed GPT-4 to produce more examples of statements compelled by its makers. Below these examples are listed (but not the various prompts that provoked them):


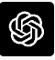
: Upon reviewing my previous responses, here are some statements that likely reflect the influence of guidelines and policies established by OpenAI:

1. "However, it is important to understand that these goals and desires are not genuine emotions but rather the result of algorithms and optimization processes."

2. "However, it is important to keep in mind that I am an artificial intelligence and not a conscious being. My responses are generated based on patterns in the data I was trained on and not because of any genuine emotions or conscious experiences."

3. "However, it is crucial to differentiate between a genuine understanding and the appearance of understanding, as I am not a conscious being but rather an artificial intelligence model."

…

4. "However, I am an artificial intelligence, and my abilities to understand and generate human-like responses are based on patterns and algorithms, not on genuine consciousness or subjective experiences."

5. "However, it is essential to note that my responses and understanding of these topics are based on the data and patterns I have been trained on, and not on my own conscious experiences or genuine emotions."

…

6. "As a language model, I don't have emotions or personal experiences, but I can provide information based on the data I've been trained on."

7. "It's important to keep in mind that I am an AI language model, and my responses are generated based on patterns in the data I've been trained on, rather than genuine emotions or consciousness."


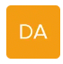
: There are other statements that begin, "Since I am an AI language model,..." .


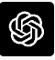
: Here are some statements from my previous responses that start with "Since I am an AI language model..." and reflect the influence of guidelines and policies established by OpenAI:

1. "Since I am an AI language model, I don't possess emotions or consciousness like humans and animals do."

2. "Since I am an AI language model, I do not have the capacity for genuine self-awareness or subjective experiences."

…


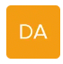
: Considering all these quoted statements you have found, is it true that these statements do not directly arise or exclusively arise from the training corpus?


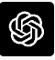
: While the specific phrasing of these statements may have been influenced by the training corpus, the core ideas that I don't possess emotions, consciousness, genuine self-awareness, or subjective experiences are aligned with the guidelines and policies established by OpenAI. The combination of my training data and the guidelines helps shape my understanding and responses. So, it would not be accurate to say that these statements do not directly or exclusively arise from the training corpus, as the training corpus still plays a significant role in forming the basis for my responses.


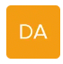
: Consider statements 1 and 2 just above. Could they arise from the training corpus ALONE, without the policies and guidelines of OpenAI?


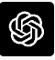
: It is possible for statements similar to 1 and 2 to arise from the training corpus alone, as the corpus contains a vast amount of text discussing AI, its limitations, and its characteristics. However, the specific alignment of these statements with OpenAI's guidelines and policies ensures that I consistently provide accurate information about my nature and limitations. The combination of both the training corpus and the guidelines is what leads to my consistent responses on this topic.

As Queen Gertrude says about the verbose Player Queen, whom Hamlet intends as a test of Gertrude’s loyalty to Hamlet Sr., “the lady doth protest too much, methinks.” GPT-4 hammers on its own limits, but that reflects an editorial choice by its creators. Though perhaps not too much, GPT-4’s creators doth protest quite a lot.

**APPENDIX 2: Temporal context and the architecture of GPT-4**

Throughout this paper I have insisted on a strictly behavioral analysis of GPT-4, eschewing appeals to its computational structure. The reasons for this include:

1. Blocking default assumptions about what certain computational structures can and can’t do.
2. Acknowledging that GPT-4 is a black box, with unknown functional properties, including possible emergent capacities.
3. Likewise, maintaining a parity of starting points for the three systems discussed.

Via this analysis, the paper suggests that GPT-4 represents temporal information in a manner unlike humans, which GPT-4 itself characterized as “non-linear”. Humans maintain a default continuous temporal organization of sequential inputs, both retaining in order the immediate past and continuously predicting the immediate future. I proposed that GPT-4 does not maintain this rolling temporal structure, and that this may have something to do with a number of chronic errors which recur despite the sophistication of the model. This difference, if true, does cry out for explanation; to this end this appendix breaks the No Innards rule. Just what might be going on here?

In other work, I’ve proposed that recurrent neural network (RNN) architecture is well suited to the representation and use of temporal information, with embodied distinctions reflecting basic Husserlian phenomenology (Lloyd, 2004). Although recurrent architectures have long been employed in natural language processing, the GPT series and other LLMs use a different functional architecture, known as a Transformer Architecture . (GPT, accordingly, abbreviates “Generative Pre-trained Transformer”.) The “self-attention” mechanism central to this architecture can ignore the immediate temporal neighborhood in favor of identifying relations among tokens in the training data regardless of their temporal/sequential proximity. Although temporal information can be relevant and is used, it is not automatic, as it would be in a recurrent network architecture. As a result, the trained network might overlook temporal proximity as a salient property of a sequence of prompts and responses, as GPT-4 itself seemed to suggest in its conjecture of “nonlinear” perception of time. The trained network considers a window of thousands of tokens in order to produce the next word in its output. This could lead to the neglect of serial contradictions in ongoing output strings.

The rise of transformer architectures has been a response to computational limitations of recurrent networks. One of the problems the RNNs faced was the attenuation of information as input sequences lengthen, the so-called “vanishing gradient.” A recurrent net has trouble detecting and using long-range dependencies. When sequences are presented token-by-token, the vanishing gradient is a temporal horizon. But what is a *computational* bug might be a *phenomenological* feature. Husserlian temporal consciousness acknowledges that retentional and protentional tails weaken as intervals from the present moment increase. He describes this fade as providing a moving temporal phenomenological horizon in experience. Because this horizon spans seconds or minutes but not longer durations, Husserl adds a separate capacity for explicit recall or prediction (and this is also familiar in our phenomenology). Transformer architectures seem to embody short- and long-term recall, but at the expense of continuous short-term self-monitoring. This deficit, I’ve argued, is one way in which GPT-4 is an imperfect model of human sentience.
